# Revealing pancrustacean relationships: Phylogenetic analysis of ribosomal protein genes places Collembola (springtails) in a monophyletic Hexapoda and reinforces the discrepancy between mitochondrial and nuclear DNA markers

**DOI:** 10.1186/1471-2148-8-83

**Published:** 2008-03-12

**Authors:** MJTN Timmermans, D Roelofs, J Mariën, NM van Straalen

**Affiliations:** 1 Department of Animal Ecology, VU University Amsterdam, Amsterdam, The Netherlands

## Abstract

**Background:**

In recent years, several new hypotheses on phylogenetic relations among arthropods have been proposed on the basis of DNA sequences. One of the challenged hypotheses is the monophyly of hexapods. This discussion originated from analyses based on mitochondrial DNA datasets that, due to an unusual positioning of Collembola, suggested that the hexapod body plan evolved at least twice. Here, we re-evaluate the position of Collembola using ribosomal protein gene sequences.

**Results:**

In total 48 ribosomal proteins were obtained for the collembolan *Folsomia candida*. These 48 sequences were aligned with sequence data on 35 other ecdysozoans. Each ribosomal protein gene was available for 25% to 86% of the taxa. However, the total sequence information was unequally distributed over the taxa and ranged between 4% and 100%. A concatenated dataset was constructed (5034 inferred amino acids in length), of which ~66% of the positions were filled. Phylogenetic tree reconstructions, using Maximum Likelihood, Maximum Parsimony, and Bayesian methods, resulted in a topology that supports monophyly of Hexapoda.

**Conclusion:**

Although ribosomal proteins in general may not evolve independently, they once more appear highly valuable for phylogenetic reconstruction. Our analyses clearly suggest that Hexapoda is monophyletic. This underpins the inconsistency between nuclear and mitochondrial datasets when analyzing pancrustacean relationships. Caution is needed when applying mitochondrial markers in deep phylogeny.

## Background

General hypotheses on arthropod phylogeny are rapidly being altered by DNA sequence data [1-3]. For instance, the Atelocerata concept held that hexapods and myriapods are united in one clade, but under the influence of molecular data (e.g. [4]) this concept was replaced by the view that crustaceans and hexapods constitute a monophyletic group, which is known as Pancrustacea (e.g. [2,3,5]).

Another recently proposed, but still highly debated viewpoint is the diphyletic origin of Hexapoda, which was initially raised by Nardi and co-workers in 2003 [6]. Based on four mitochondrial genes, they [6] observed that two species of Collembola (*Tetrodontophora bielanensis *and *Gomphiocephalus hodgsoni*) branched off before the other pancrustacean groups that were included in their study (Insecta and Crustacea), suggesting paraphyly of Hexapoda. Their thesis was that the six-legged body plan of Collembola and other hexapods evolved at least twice: once in the group of wingless hexapods and another time in the true insects.

The conclusions of Nardi et al. [6] resulted in a vivid scientific debate, and many studies have addressed the phylogenetic placement of Collembola since then. Some authors focused on mitochondrial sequences, others analyzed nuclear genes. Additional mitochondrial sequences confirmed that, due to the placement of Collembola separate from the other hexapods, Hexapoda are indeed not monophyletic [3,7]. However, after thorough analyses exploring the effects of outgroup and gene choice, sequence handling and optimality criteria on inferred trees, Cameron and co-workers [8] concluded that the mitochondrial data as available at the time were inadequate to fully resolve hexapod relationships [8]. Hassinin [9] arrived at a similar conclusion in a more recent study focusing on the effects of reverse strand-bias. Most recently, Carapelli and co-workers [10] reported new analyses on a very large dataset, consisting of no fewer than a hundred almost-complete mitochondrial genomes. These new analyses, which were based on a novel model of amino acid sequence evolution (MtPan), supported the non-monophyly of hexapod groups.

It has gradually become clear in pancrustacean phylogeny that nuclear and mitochondrial datasets tell different stories, and often result in different conclusions [10]. Remarkably, studies that addressed the question using nuclear genomic data (ribosomal RNA and protein-encoding genes) indicate that the Collembola group between crustaceans and insects and that Hexapoda is monophyletic [2,5,11-18]. However, most of those studies included a relatively small number of loci [2], most likely because obtaining data on protein-encoding DNA sequences is not always straightforward for groups for which little genomic information is available. Here we try to fill this gap by re-evaluating the position of Collembola using a relatively large number of nuclear protein-encoding sequences that are, although all for ribosomal proteins, assumed to be distributed throughout the genome (see for example [19]).

Several authors have shown that publicly available data can be useful when conducting a large-scale phylogenetic study (eg. [20]), and that expressed sequence tags (ESTs) can be extremely valuable for phylogenetic purposes [21,22]. Here, we combine data from a recently finished EST sequencing project on the collembolan *Folsomia candida *[23], with data on 34 ecdysozoan species (Chelicerata, Hexapoda, Tardigrada, Nematoda and Crustacea) available in the public GenBank repository [24], and with data from a smaller EST dataset of the collembolan *Orchesella cincta*. We focus on ribosomal proteins to prevent the problem of analyzing paralogous genes (*sensu *[21]).

## Results

In total, gene-sequences for 48 ribosomal proteins were obtained from the *Folsomia candida *EST dataset. This is almost two-thirds of the total set of 79 ribosomal proteins [19] found in the genome of *Drosophila melanogaster*. Four *D. melanogaster *ribosomal protein sequences (RpL15, RpL32, RpL36 and RpL39) showed high similarity with two, instead of one *F. candida *transcript cluster in the EST dataset. Comparison of the *F. candida *transcripts with those of *D. melanogaster *revealed insertions/deletions resulting in frame shifts in one of the two *F. candida *EST clusters for RpL15, RpL32 and RpL36. Transcripts with a frame-shift were discarded. Two highly diverse *F. candida *EST clusters (one consisting of six EST sequences and one singleton sequence) showed homology with *D. melanogaster *RpL39. The *F. candida *RpL39 singleton sequence was excluded from further analysis. The discarded RpL15, RpL32, RpL36 and RpL39 transcripts may stem from duplications in the *F. candida *genome (for example, in *D. melanogaster *nine ribosomal proteins are represented by two separate functional genes [19]), or from constitutively expressed pseudogenes. This situation may be analogous to the apparent amplification of many mammalian ribosomal proteins; for instance, the human genome contains over 2000 ribosomal protein pseudogenes [25]. Still, it seems that only one copy of each ribosomal protein is actually functional [26,27].

As described in the methods section, the remaining 48 ribosomal protein sequences were used to retrieve ribosomal protein sequence information on 32 additional ecdysozoan species. In addition, ribosomal protein sequences of *D. melanogaster, Apis mellifera *and *Caenorhabditis elegans *were retrieved from the Ribosomal Protein Gene-database (RPG; [28]). The number of usable (partial) ribosomal protein gene sequences that were obtained per species ranged from two (4% of the 48 genes: *Amblyomma variegatum*) to 48 (100% of the 48 genes: *D. melanogaster *and *Apis mellifera*) (Table 1). Redundancy for a given ribosomal protein gene in a given species was often low, and many gene sequences were represented by one or a few EST sequences only. It should be mentioned that due to this rather low sequence coverage the dataset is most probably not free from sequencing errors. Furthermore, none of the 48 ribosomal proteins that were included in the dataset were observed in all of the 36 species investigated (Table 1 and Additional file 1). In summary, for each ribosomal protein gene information was available for 25% to 86% of the taxa.

**Table 1:** Ribosomal protein-sequences and species included

Ribosomal protein	Length in alignment	# of variable sites	Occurrence*	Clade	Species	Common name	Occurrence***
RpL32	134	110	31	Nematoda	Caenorhabditis elegans	Roundworm	47
RpL11	133	71	29	Tardigrada	Hypsibius dujardini	Water bear	38
				
RpL13A	146	108	29	Collembola	Folsomia candida	Springtail	48
RpS8	34	15	29		Orchesella cincta	Springtail	7
				
RpS5	150	68	28	Insecta	Apis mellifera	Honeybee	48
RpS6	98	58	28		Drosophila melanogaster	Fruit fly	48
RpS7	168	128	28		Locusta migratoria	Migratory locust	47
RpL21	164	120	27		Acyrthosiphon pisum	Pea aphid	45
RpL24	52	40	27		Tribolium castaneum	Red flour beetle	44
RpS15	141	85	27		Plutella xylostella	Diamondback moth	44
RpS18	116	56	27		Toxoptera citricida	Brown citrus aphid	40
RpS23	140	64	27		Manduca sexta	Tobacco hornworm	38
RpL12	150	81	25		Culicoides sonorensis	Mosquito	36
RpL13	39	32	25		Glossina morsitans	Tsetse fly	35
RpL31	108	87	25		Ctenocephalides felis	Cat flea	34
RpS11	151	104	25		Homalodisca coagulata	Glassy-winged sharpshooter	32
RpS12	106	83	25				
RpS16	137	73	25		Pediculus humanus	Human head/body louse	22
RpS9	130	49	25				
RpL15	169	117	24		Diaprepes abbreviatus	Root weevil	21
RpS14	152	58	24		Tricholepisma aurea	Silverfish	18
RpS19	130	107	24		Ips pini	Pine engraver	16
RpL34	93	73	23		Anopheles funestus	African malaria mosquito	11
RpL37A	87	43	23				
RpLp0	36	32	23	Crustacea	Daphnia magna	Water flea	44
RpS26	103	45	23		Litopenaeus vannamei	Pacific white shrimp	44
RpL10A	42	36	22				
RpL23	139	51	22		Penaeus monodon	Black tiger shrimp	43
RpL36A	105	44	22		Litopenaeus setiferus	Northern white shrimp	36
RpL39	51	26	22				
RpS17	112	70	22		Homarus americanus	Atlantic lobster	30
RpS27	80	42	22		Marsupenaeus japonicus	Kuruma shrimp	25
RpS30	89	72	22		Artemia franciscana	Brine shrimp	14
RpL36	87	64	21		Callinectes sapidus	Blue crab	13
RpL35	127	68	20		Eurydice pulchra **	Speckled sea louse	4
				
RpL35A	78	58	20	Chelicerata	Amblyomma americanum	Lone star tick	32
RpL37	80	43	20		Boophilus microplus	Southern cattle tick	29
RpL3	50	28	20		Rhipicephalus appendiculatus	Brown ear tick	21
RPL40****	128	22	20				
RpS29	56	30	20		Ornithodoros porcinus	Tick	19
RpS15A	80	33	19		Sarcoptes scabiei	Scabies mite	13
RpL38	70	37	17		Amblyomma variegatum **	Tick	2
RpS21	86	49	17				
RpS28	66	18	17				
RpL27	137	84	14				
RpLp2	83	59	12				
RpS27A****	152	27	12				
RpL22	69	23	9				

Left: The inferred ribosomal protein sequences (amino acids) that were included in the concatenated dataset. Only ribosomal proteins present in the *Folsomia candida *EST dataset were included in the analysis. Occurrence*: The number of species for which was data available for a certain ribosomal protein. # of variable sites: positions that constitute more than one amino acid in the different alignments. Numbers were calculated in MEGA4 [61].

Right: All species that were included in the analyses. Species marked with ** were excluded from the final analyses because they contained too few sequences. Occurrence***: The number of genes (out of 48) that were available for a specific taxon. ****: RpS27a and RpL40 are fused to ubiquitin [25].

**Figure 1:**
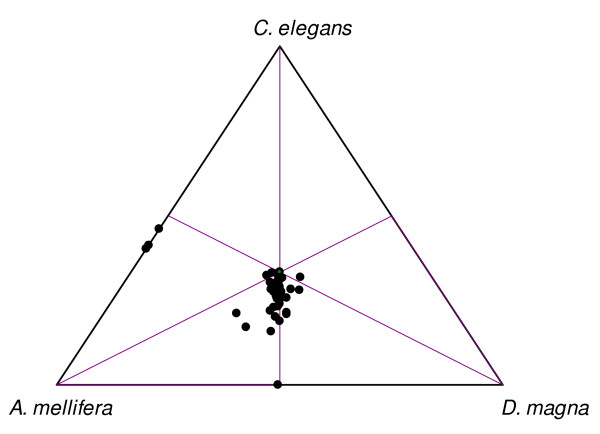
**Ternplot showing "similarity' between *Folsomia candida *and *Apis mellifera, Daphnia magna *and *Caenorhabditis elegans*, respectively.** Each dot represents one ribosomal protein. The four dots that are visible on the three edges represent five genes that were unavailable for one of the three species. Two dots/genes overlap. RpS30 was not mapped on this graph, as analysis of our RpS30 alignment resulted in a Kimura protein distance that was larger than one: Kimura protein distance *C. elegans *and *F. candida *= 1.14.

We calculated "similarity" values between the amino acid sequences of *F. candida *and three well-represented species (*C. elegans *(most-distant outgroup), *Daphnia magna *and *A. mellifera*). These values were mapped onto a ternary graph (Figure 1). Almost all points cluster in the lower region of the ternary graph, showing that for almost all genes the distance between *F. candida *and *C. elegans *is greater than the distance between *F. candida *and *A. mellifera*. The graph also shows that most genes of *F. candida *are more "similar" to *A. mellifera*, while some have more in common with *D. magna*.

The individual alignments were concatenated and phylogenetic analyses were conducted to investigate the position of Collembola. Two species were excluded from the analyses (Table 1). The final alignment had a length of 5034 inferred amino acids, representing in total 15,102 nucleotides. Information was available for 66% of the amino acid positions.

Likelihood mapping was applied to obtain estimates of phylogenetic signal. The concatenated dataset contained more phylogenetic signal (89% fully resolved quartets) than each of the independent ribosomal protein alignments (9–72% fully resolved quartets; data not shown).

**Figure 2:**
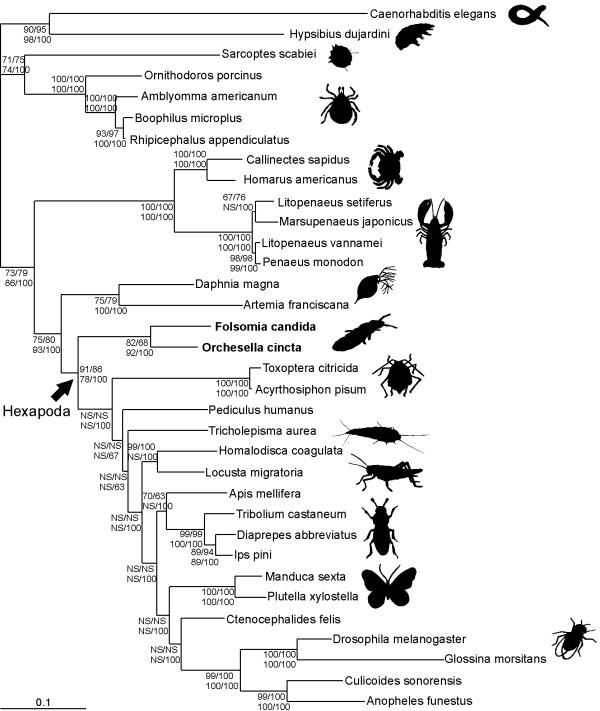
**Topology based on the Bayesian analysis (conducted in software package MrBayes [59]).** All other phylogenetic reconstructions were highly comparable, except for several differences within the Insecta. NS: Not supported. Numbers at each node show bootstrap support or posterior probabilities: $\begin{array}{l} \text{Bootstrap ML RtREV+G+F / Bootstrap ML WAG+G+F}\\ \text{Bootstrap MP / Bayesian posterior probabilities RtREV+G+F} \end{array}$

The trees obtained by the different tree-reconstruction algorithms were highly comparable (Figure 2). In all reconstructions (MP, ML and Bayesian), Chelicerata and Pancrustacea each formed a monophyletic group, with relatively high support (Bayesian posterior probabilities both 100%). The two branchiopods included in this study (*D. magna *and *Artemia franciscana*) grouped together, and remained separate from the other crustaceans (Malacostraca).

The relationships within the Insecta were weakly resolved; however, Diptera was recovered as a monophyletic clade, as were Lepidoptera and Coleoptera (Figure 2). However, the Hemiptera were resolved as a paraphyletic group. *Homalodisca coagulata *grouped with *Locusta migratoria *(Orthoptera), rather than with the other hemipterans *Acyrthosypon pisum *and *Toxoptera citricida*. The highly supported, but obviously incorrect, positioning of *Homalodisca coagulata *does not seem to be an artefact of the method that allowed for missing data, since all three Hemiptera, as well as *Locusta migratoria*, were represented by a large number of ribosomal protein gene sequences (32 to 47). The incorrect placement of *H. coagulata *could be a consequence of the inability of ribosomal protein genes to resolve more recent evolutionary splits, which may be a trade-off of their suitability for deeper phylogenies.

Hexapoda was clearly monophyletic: Both collembolans (*F. candida and O. cincta*) grouped together and formed the sister-group to the Insecta in all analyses conducted (ML bootstrap RtRev+G+F = 91%, ML bootstrap Wag+G+F = 86%, MP bootstrap = 78%, Bayesian posterior probabilities = 100%).

Several *C. elegans *and *D. melanogaster *ribosomal proteins are duplicated (see also RPG database). MP analyses of a second concatenated dataset that contained *D. melanogaster *homologs for ribosomal protein RpS5, RpS15A, RpS19, RpS28, RpL34 and RpL10A resulted in a similar topology (data not shown).

## Discussion

In this study we reassessed the position of Collembola, using (partial) genes for 48 nuclear encoded proteins. The main result of our study is clear evidence of monophyly of Hexapoda. All phylogenetic reconstruction methods employed in this study support this hypothesis (Figure 2). Based on our nuclear dataset we conclude that the six-legged body plan, as found among insects and Collembola, evolved only once in the course of evolution. This is in contrast to results obtained using large mitochondrial datasets [3,6,9,10] that by and large suggest that the characteristic hexapod body plan was acquired in parallel by Collembola and insects due to convergent evolution, rather than by descent.

Discrepancies between pancrustacean relationships as revealed by either nuclear or mitochondrial datasets seem almost universal. It is of major importance to focus on the causes of these discrepancies, and whether or not one of the two types of markers is superior. Elaborate discussions on the 'pros and cons' of one or both of the two different markers, and the possible approaches on how to correct for ambiguous signals are given in several recent papers [8,9,29-32]. Comparative studies that contrast nuclear and mitochondrial datasets suggest that nuclear markers are preferred in deep arthropodan molecular phylogenetics, as mitochondrial genes tend to be more substitutionally biased and evolve (in general) in a much faster way [30].

Already in 1999 Curole and Kocher [33] stated in a review paper that the value of mitochondrial genes in deep-level phylogeny is debatable and that "controversial" mitochondrial DNA (mtDNA) results should be verified with nuclear encoded genes. This was also the final conclusion of Springer and co-workers [34]. These authors compared the usability of nuclear and mitochondrial encoded genes in inferring deep-level mammalian phylogenies. The authors report that nuclear encoded genes (exons) outperform mitochondrial markers in resolving deep splits. Springer and co-workers suggest that the reason for this dissimilarity in resolving-power might be found, among others, in the rate of nucleotide substitution [34].

Still, although the nuclear protein-encoding sequences in the study of Springer et al. [34] outperformed the mitochondrial genes, mtDNA-based studies are not necessarily useless for deep phylogeny. They are only problematic if mitochondrial genomes evolve at such a rate that saturation of substitutions makes actual phylogenetic signals from deeper nodes hazy [35]. Otherwise, analyses using appropriate models should still be able to retrieve a plausible tree [35]. In a recent study, Kjer and Honeycutt [35] used an approach that included all data found in mitochondrial genomes (including for instance 3^rd ^codon positions, but excluding the control region). After applying a site-specific rate model, these authors retrieved a phylogenetic tree of mammals that was in accordance with recent nuclear DNA based phylogenies [35].

When investigating cheliceratan relationships Jones et al. [36] arrived at a comparable conclusion. These authors state that mtDNA can be applied in molecular phylogenetics, but only when an appropriate substitution model (e.g. to correct for strand-bias) is used. These authors state as a final remark that earlier mtDNA studies that focused on deep-phylogenetic questions should be thoroughly re-evaluated [36]. However, such models of mitochondrial sequence evolution might first need to be developed before Collembola can be placed with certainty in the arthropod phylogenetic tree. As mentioned before, Carapelli and co-workers [10] investigated an innovative pancrustacean-model of mitochondrial protein change. This model significantly aided the tree building, but did not yield a monophyletic Hexapoda [10].

An advantage of ribosomal protein genes is that the sequences of different species can be relatively easily homologized due to their conserved nature. However, there are also disadvantages. Although ribosomal protein genes are distributed all over the genome, they definitely do not evolve independently. Coevolving sites are known to exist in ribosomal proteins [37]. For example, amino acid residues that are near tRNA binding sites in the ribosome appear to evolve in a related manner [37].

It has to be mentioned that we included only two Collembola in our analyses. Preferably, more springtail species, and maybe even more importantly, proturans and diplurans, should be included. Those latter basal hexapod groups were excluded from the current analysis as they lack available (EST) data. While earlier work suggests that proturan and dipluran genes might be fairly divergent from other arthropods [11], this and other papers (e.g. [21,22]) suggest that it should be relatively easy to obtain phylogenetically relevant sequence information on those groups by EST sequencing.

Another intriguing result of this study is the non-monophyly of the crustaceans. The branchiopods *D. magna *and *A. franciscana *clustered with the hexapods rather than with the other crustaceans in the malacostracan group. This is in accordance with studies by Regier and co-workers [5] and Mallat and Giribet [12], which suggests that the hexapod lineage evolved from within the crustaceans [38]. The observed close relationship between hexapods and branchiopods, in combination with some other characteristics, made Glenner and co-workers [38] suggest that branchiopod groups colonized terrestrial ecosystems as insects.

As a final remark we would like to point out that this study shows that Collembola occupy a crucial position. Obtaining additional (EST) sequence information on Collembola, as well as other basal hexapods (Protura, Diplura and Microcoryphia) will definitely result in a better understanding of the phylogenetic origin of insects.

## Conclusion

The phylogenetic efforts presented here clearly show that Collembola is a sister group of Insecta (Figure 2). Our results reinforce the discrepancy between results obtained using mitochondrial and nuclear datasets. It seems of major importance to unravel the underlying causes of the disagreements observed, or otherwise focus on nuclear encoded genes.

## Methods

### EST dataset and ribosomal protein selection

Recently, approximately 9.000 *F. candida *EST sequences were generated (see [23] for additional information). In order to select springtail ribosomal protein gene sequences from this EST dataset ribosomal protein cDNA sequences of *Drosophila melanogaster *were retrieved from the Ribosomal Protein Gene database (RPG [28]). These sequences were then compared with the *F. candida *EST dataset using TBlastX [39]. Springtail sequences showing significant similarity (E value < 10^-10^) were used for further analysis. All *F. candida *sequences are stored in dbEST.

### Sequence retrieval and DNA alignment

For this study 35 additional species, comprising nineteen hexapod species, nine crustacean species, five chelicerates, and two non-arthropod ecdysozoans (one nematode, and one tardigrade) were selected (Table 1). For 31 species all available nucleotide sequences were retrieved from NCBI Genbank (including ESTs) using a Perl script, BioPerl [40] and NCBI Entrez Programming Utilities [41]. Species-specific BLAST databases were constructed. The *F. candida *ribosomal protein gene sequences, obtained as described above, were compared to these databases using TBlastX (minimal E value < 10^-10^). For every species, the sequences showing significant resemblance to a specific ribosomal protein were retrieved using Perl and BioPerl [40] and grouped in a FASTA file (with a maximum of 24 sequences per ribosomal protein per species). Additional file 2 shows all GenBank accessions that were used. The software program Phrap (P. Green, pers. comm. [42]) was applied to assemble a "consensus" sequence for each of these FASTA files: Phrap combines all available sequences and takes sequence coverage into account, which results in more precise consensus sequences. When Phrap created more than one sequence for a given ribosomal protein gene in a given species, the sequence part that was most abundant in the original sequence dataset was used for further analysis. All obtained nucleotide sequences were automatically translated to high quality peptides using the software program prot4EST [43].

The 48 ribosomal sequences were in addition compared to three smaller and unpublished collembolan (*Orchesella cincta*) EST datasets. These *O. cincta *ESTs were generated from libraries constructed by Roelofs and co-workers [44], Ellers and co-workers [45] and T.K.S Janssens. Finally, *D. melanogaster*, *C. elegans *and *Apis mellifera *protein sequences were obtained from RPG as well.

For each ribosomal protein gene, the protein or the prot4EST inferred amino-acid sequences of the different species were aligned using ClustalW [46] and inspected with GeneDoc [47]. If for a certain species a ribosomal protein was represented by more than one locus in the RPG database, one ribosomal protein was randomly taken. Additional alignments were made in which the chosen *D. melanogaster *sequences were replaced by their homologous counterparts. This was done for RpS5, RpS15A, RpS19, RpS28, RpL34 and RpL10A. Sequences that aligned poorly were subjected to visual inspection, and those sequences that appeared to be out of frame from an identifiable amino-acid position were manually corrected and re-aligned. This implied that insertions and deletions causing frame-shifts were characterized as missing or were removed. All alignments were trimmed to the length of the *F. candida *sequence. Finally, inadequately aligned regions were excluded from further analysis using the program Gblocks [48].

### Phylogenetic analysis

First, to obtain insight into the information contained by each of the 48 inferred ribosomal protein sequences, the distances (Kimura's distance [49]) between *F. candida *and three well-represented species (*C. elegans *(outgroup), *D. magna *(Crustacea) and *A. mellifera *(Insecta)) were calculated using the PHYLIP package Protdist [50]. Those values were used to calculate "similarity" values by subtracting the distance value from one (similarity = 1 - distance). Similarities were visualized in a ternary graph in Microsoft Excel, using TernPlot [51]. All the individual alignments were additionally subjected to a likelihood mapping analysis using Tree-Puzzle [52,53] (max. 10.000 quartets, WAG model [54] of substitution) in order to assess the phylogenetic signal in the dataset.

Second, all the individual alignments were concatenated into a single alignment. If due to the presence of paralogous *D. melanogaster *sequences two alignments were available for one ribosomal protein, only one was included. This procedure resulted in a dataset with spaces of missing data (*sensu *[20]). The alignment is available from [55]. To check if the final outcome depended critically on the choice for one or the other paralog, a second concatenated alignment was made in which each *D. melanogaster *homolog was replaced by its counterpart (for RpS5, RpS15A, RpS19, RpS28, RpL34 and RpL10A).

The first concatenated dataset was analyzed with Tree-Puzzle [52] as described above. Subsequently, this alignment was analyzed with Maximum Parsimony (MP), Maximum Likelihood (ML) and Bayesian methods. ML analyses (100 bootstrap replicates) were conducted using the Linux version of Phyml v2.4.4 [56], applying substitution models that were selected with ModelGenerator (gamma distribution with four rate categories)[57]. The selected model for the translated dataset was RtREV+G+F [58]. The ML analysis of the inferred amino acid dataset was repeated using the WAG+G+F substitution model; this model is appropriate for soluble proteins like ribosomal proteins [21], and provided the third-best data fit after the RtREV+G+F and the RtREV+I+G+F models. Bayesian analysis (RtREV+G+F) was conducted using the Windows version of MrBayes [59]. Analyses were run for 1,000,000 generations (MCMC sampling without heating, "one chain" and tree-sampling every 100 generations). The log likelihood values for the different generations were used to determine stationarity by plotting them, and the first 50,000 generations were discarded as "burn-in".

The ML and Bayesian analysis used the same model of sequence evolution for all the concatenated genes. However, likelihood methods restricted to only one model might perform inadequately when analyzing concatenated datasets [60]. Therefore, the data was analyzed using Maximum Parsimony, which might address this problem. The MP analysis was performed in the software MEGA [61] under Windows using all available sites (1,000 bootstrap analyses; Starting tree obtained by Random Addition). The second concatenated dataset, which contained the homologous counterparts of duplicated *D. melanogaster *ribosomal proteins was analyzed using MP only.

Bootstrap values above 70% (ML and MP) or 95% (Bayesian) were deemed significant.

## Authors' contributions

MJTNT, DR and NMvS designed the study. MJTNT conducted the bioinformatic and phylogenetic analyses and wrote the manuscript. JM generated *Orchesella cincta *EST data. DR, NMvS and JM commented on the manuscript. All authors read and approved the last version of the manuscript.

## Supplementary Material

Additional file 1 Representation of inferred amino acid sequence overlap between the different species in the concatenated alignment. Click here for file

Additional file 2 GenBank accession numbers of entries used to construct the concatenated alignment. Click here for file
